# Zearalenone and Its Masked Forms in Cereals and Cereal-Derived Products: A Review of the Characteristics, Incidence, and Fate in Food Processing

**DOI:** 10.3390/jof8090976

**Published:** 2022-09-18

**Authors:** Huilin Yu, Junhui Zhang, Yixuan Chen, Jiajin Zhu

**Affiliations:** Department of Food Science and Nutrition, Zhejiang University, Hangzhou 310029, China

**Keywords:** zearalenone, cereal and cereal-based food, characteristic, incidence, food processing

## Abstract

Zearalenone (ZEA) is known as a Fusarium-produced mycotoxin, representing a risk to cereal food safety with repercussions for economies and worldwide trade. Recent studies have reported the co-occurrence of ZEA and masked ZEA in a variety of cereals and cereal-based products, which may exert adverse effects on public health due to additive/synergistic interactions. However, the co-contamination of ZEA and masked ZEA has received little attention. In order to minimize the threats of co-contamination by ZEA and masked ZEA, it is necessary to recognize the occurrence and formation of ZEA and masked ZEA. This review focuses on the characteristics, incidence, and detection of ZEA and its masked forms. Additionally, the fate of ZEA and masked ZEA during the processing of bread, cake, biscuits, pasta, and beer, as well as the ZEA limit, are discussed. The incidence of masked ZEA is lower than that of ZEA, and the mean level of masked ZEA varies greatly between cereal samples. Published data showed a considerable degree of heterogeneity in the destiny of ZEA during cereal-based food processing, mostly as a result of the varying contamination levels and complicated food processing methods. Knowledge of the fate of ZEA and masked ZEA throughout cereal-based food processing may reduce the likelihood of severe detrimental market and trade ramifications. The revision of legislative limits of masked ZEA may become a challenge in the future.

## 1. Introduction

Cereal and cereal-based products constitute the majority of the world’s dietary energy and nutrients, accounting for more than half of the average per capita caloric consumption and nearly half of the average per capita protein intake [1,2]. Cereal grains have a long history in the brewing industry as raw ingredients. They can also be processed into flours that are utilized in the production of bread, pasta, cake, and biscuits [3,4]. Additionally, cereal by-products play a crucial function in animal feeding because they supply the required energy and protein for the growth of livestock. It was estimated that approximately 2799 million tons of cereal will be produced in 2021, and cereal production has a significant impact on global food security [5].

However, cereals are easily contaminated with fungi during planting, resulting in mycotoxin production [6]. Mycotoxins are the toxic secondary metabolites of fungi associated with adverse effects on animals, humans, and crops, leading to health issues and economic losses [7]. The major mycotoxins with agro-economic importance are aflatoxin, fumonisins, ochratoxin, zearalenone (ZEA), and trichothecenes, and ZEA is one of the most widespread mycotoxins in cereal [7,8]. ZEA is generated by *Fusarium* genera such as *F. graminearum* (*Gibberella zeae*), *F. culmorum*, *F. crookwellense*, *F. poae*, *F. semitectum*, and *F. equiseti* [9,10]. After plant and fungal conversion, animal metabolism, and matrix effects and reactions during food processing, ZEA can be transformed to masked ZEA in cereal and cereal-derived commodities [11,12,13]. Concerns about the safety of contaminated products have been further heightened by these masked ZEA. Due to analytical challenges, masked ZEA may not be detectable in cereals, posing potential threats in vivo for their retained toxicity.

Given the prevalence of ZEA and masked ZEA in cereals, consumption of contaminated cereal and cereal-derived products may comprise a significant source of ZEA exposure in humans [14]. In implementing preventative strategies to lower the intake risks of ZEA and masked ZEA, it is of utmost significance to not only know the characteristics of ZEA and masked ZEA in cereals and final commodities but primarily the fate of ZEA in typical cereal-derived food processing, such as bread, cake, biscuit, pasta, and beer.

## 2. Characteristics and Biosynthesis of ZEA and Masked ZEA

### 2.1. ZEA

**Figure 1:**
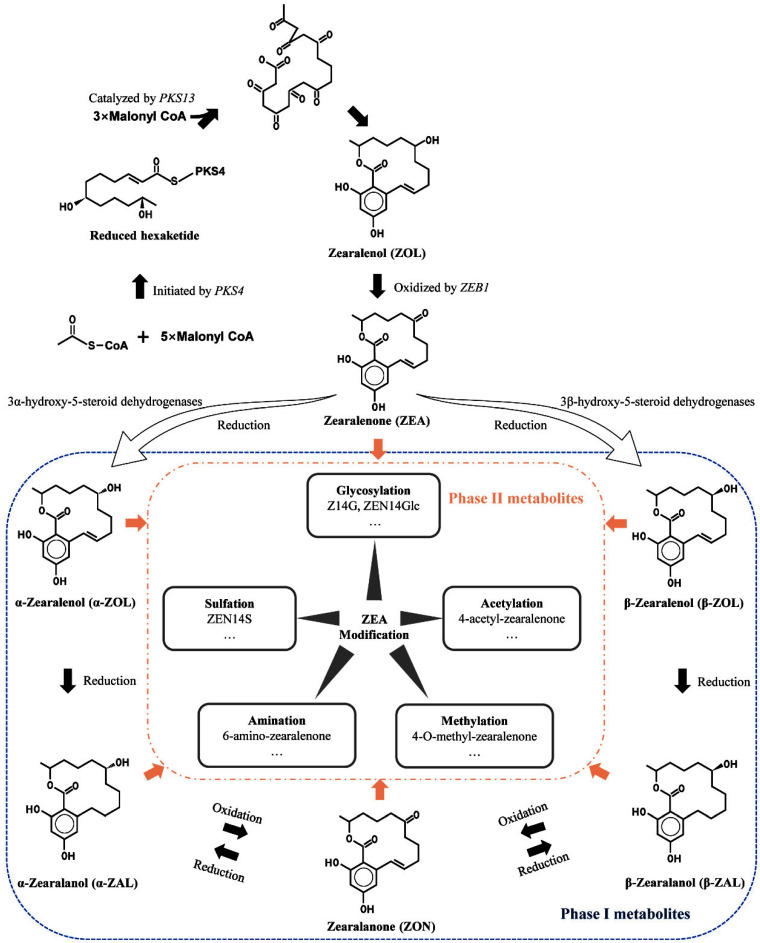
An overview of ZEA and masked ZEA formation.

ZEA, C_18_H_22_O_5_ (Figure 1), with a molar mass of 318 g/mol, existing as white crystals, can dissolve in alkaline aqueous solutions and some organic solvents such as acetone and ethanol, but is insoluble in water [15]. ZEA exhibits high thermal stability (it is stable up to 150 °C) [16]. ZEA in cereals is produced by *Fusarium* species, particularly *Fusarium graminearum* and *Fusarium culmorum*. Synthesis of ZEA in cereal foods relies on several factors, such as water activity (a_w_), temperature, incubation time, the composition of storage atmosphere, cultural practices, pH, food substrate, mold abundance, and the effect of anti-fungal agents [17,18]. These factors have a complicated influence on ZEA production, making it difficult to obtain general conclusions about optimal conditions for ZEA production that can be applied to different fungal strains to control ZEA production [18]. Further studies are needed to illustrate the relationship between fungal growth and ZEA production and its molecular mechanisms under the impact of several factors.

Years of research have figured out the synthesis pathways of ZEA [19,20,21,22,23]. Generally, polyketide ZEA is from the oxidation of zearalenol (ZOL), which acts as the precursor deriving from the head-to-tail condensation of nine acetate units via the acetate–polymalonate pathway. Polyketide synthases (*PKS*s), *ZEB* genes, and other clustered regulatory genes participate in synthesizing ZEA. A scheme for ZEA synthesis is presented in Figure 1. *PKS4* plays a part in initiating ZEA biosynthesis by encoding an enzyme to drive carbon condensation from CoA to form hexaketide. The non-reducing polyketide synthase gene *PKS13* catalyzes malonyl-CoA’s connection to the pre-formed reduced hexaketide and folding of non-reduced ketide units. *ZEB1* was reported to take part in ZOL oxidation to ZEA via encoding isoamyl alcohol oxidase, and *ZEB2* regulated transcription of other genes, which was primarily affected by nutrient and pH conditions [19]. Gaffoor et al. [20] also confirmed that *ZEA1* and *ZEA2* were indispensable in ZEA production, mainly responsible for carbon addition. Though various *PKS*s of *Fusarium* species have been identified, studies on their functions are still scarce.

### 2.2. Masked ZEA

Masked ZEA refers to zearalenone derivatives produced through a variety of processes. They can be precursors, metabolites, or degradation products of the “parent” (or free) form of the ZEA, or they can be the results of a biotic or chemical reaction between ZEA and the matrix [24,25]. *Fusarium graminearum* is capable of producing ZEA-sulfate, α-zearalenol (α-ZOL), and β-zearalenol (β-ZOL) [26]. *Fusarium* and other fungal co-contaminations enhance the likelihood of masked ZEA incidence. In addition, microbial biotransformation, plant response, in vivo metabolism, and food processing are common methods of masked ZEA formation. *Rhizopus arrhizus* could transform ZEA to ZEA-4-O-sulfate [27]. When co-incubated with *Aspergillus* and *Rhizopus*, ZEA was found to convert to several conjugated forms, such as ZEA-14-sulfate (ZEA14S), ZEA-O-14-glucoside (ZEA14Glc), ZEA-O-16-glucoside (ZEA16Glc), α-ZOL, and α-ZOL-sulfate [28]. Plant responses to ZEA modification are considered their defense against xenobiotics during growth. In plants, the modified ZEA (biologically modified) would typically undergo compartmentalization into the vacuole and the cell wall [29]. Diverse plant defenses may enhance the unpredictability of food processing and oral consumption. In vivo metabolism of ZEA in animals can be regarded as a detoxification process, through which ZEA is modified and finally eliminated through urine and feces [30]. ZEA in food can be converted to masked ZEA for complex chemical and biological transformation in food processing.

Although masked ZEA can be generated in multiple ways, the modified ZEA is generally classified into two categories, phase I and phase II metabolites (Figure 1). Phase I conversion means that free ZEA is oxidized, reduced, or hydrolyzed, such as α/β-ZOL, α/β-zearalanol (α/β-ZAL), and zearalanone (ZON). It is usually deemed as a transforming process to higher toxicity for ZEA because of the more estrogen-like structure alteration. Phase II conversion, during which ZEA or phase I metabolites are conjugated with endogenous molecules such as sugars, amino acid, acetic, and sulfate, is regarded as a detoxification process. During these courses, more hydrophilic compounds are synthesized, facilitating the elimination of mycotoxins.

## 3. Incidence of ZEA and Masked ZEA in Cereal and Cereal-Based Food

ZEA contamination in cereal and cereal-based food has been a long-lasting safety issue. ZEA can be considered as a widespread mycotoxin contaminant, despite its relatively low concentration in the majority of cereals. Research for thirty samples of maize, wheat, oats, and other cereal-derived food revealed an 80% incidence of ZEA [31]. The World Health Organization [32] studied ZEA incidence in crops around the globe and reported that approximately 30–40% of crops are contaminated with ZEA. It was proposed that the rate of ZEA contamination ranked second in cereals and cereal-based food samples, with about 46% of barley as well as 24% of wheat products being contaminated with ZEA [33]. A survey from the EFSA Panel on Contaminants in the Food Chain [34] showed that the frequency of ZEA occurrence in maize reached 33%, with the mean level reaching 15 μg/kg. ZEA occurrence in maize germ oil occupied a higher percentage of 86%, and the mean content was as high as 72 μg/kg. Lee et al. [35] compiled the global occurrence data over the past 10 years and presented that the incidences and maximum levels of ZEA in raw cereal were 46% and 3049 μg/kg, respectively.

The prevalence of ZEA contamination in cereals depends on several factors, e.g., the sample differences, weather fluctuations, and processing [36]. According to published works, grains and animal feed are the items most frequently exposed to ZEA [37,38]. Temperatures that are mild or low are more favorable for ZEA production. Previous work demonstrated that fungi stressed between 8–25 °C were able to produce ZEA [39,40], while ZEA was not generated over 37 °C [41]. The effects of humidity on the development of fungi and mycotoxin were shown to be larger than that of temperature. *Fusarium* species typically produced ZEA under conditions with a water activity greater than 0.9 [42]. A 10-year global survey suggested that ZEA concentration was positively correlated with precipitation proportion [43]. Stanciu et al. [44] found that ZEA registered the highest frequency with medium-to-high precipitation and moderate temperatures. In addition, a higher CO_2_ concentration in the atmosphere increased the pathogenicity of fungi and the susceptibility of crops to pathogens [45]. Some pre-harvest and post-harvest procedures, such as rotation, weeding, drying, and hulling, affected the ZEA level in cereals. The persistence of fungi on leftover debris resulted in successive infestation during rotation [46]. Weeds could be alternative hosts for the *Fusarium* species complex, causing ZEA contamination in cereals [47]. The practices that influenced ZEA existence during processing are discussed in detail in Section 5.

Masked ZEA was also present in cereal and cereal-based foods, although it was not as prevalent as ZEA. Table 1 provides an overview of the incidence of masked ZEA in cereals and cereal-based food. The incidence and mean level of masked ZEA vary greatly among different cereal samples. The phase I metabolites, primarily β-ZOL and α-ZOL, were broadly distributed and rather abundant in cereals. Comparatively, a variety of phase II metabolites were found in several cereals, including maize, wheat, oats, and barley. It is quite probable that cereals contain additional phase II metabolites. It is important to note that some masked ZEA was present in cereal-based food stuffs with high concentrations (e.g., β-ZOL and α-ZOL in bread reached 79 µg/kg and 64 µg/kg, respectively [31]). In light of the possible toxicity of masked ZEA, more data on masked ZEA occurrences in cereal and cereal-based food should be collected to facilitate risk assessment and hazard control of masked ZEA.

## 4. Methods of Detection

Chromatographic, immunochemical, and electrochemical detection methods, such as liquid chromatography–tandem mass spectrometry, enzyme-linked immunosorbent assay, and electrochemical biosensors, are extensively employed for the detection and quantification of ZEA [50,51,52,53,54,55].

LC-MS/MS is the most used method for ZEA and masked ZEA detection. The instrumental manner typically includes sample preparation, extraction, sample pretreatment, separation, and determination. Vendl et al. [56] developed an LC-MS/MS method with recoveries above 70% for monitoring the levels of ZEA, zearalenone-4-glucoside, α-ZOL, β-ZOL, α-zearalenol-4-glucoside, and β-zearalenol-4-glucoside in four cereal-based food matrices. This method performs well in terms of accuracy, precision, and repeatability, but has limits for large-scale and on-site rapid analysis due to its complex analytical methodologies, high cost, and need for analysts. Moreover, despite the fact that several masked ZEAs, such as α/β-ZOL, zearalenone-4/14/16-glucoside, and zearalenone-4/14-sulfate, were found in cereal samples using LC-MS/MS, analysis for masked ZEA remains challenging because of the occurrence of several unknown forms of masked ZEA and the lack of analytical standards and accurate LC-MS/MS methods [56].

Immunoassay can evaluate multiple food samples concurrently for masked ZEA detection, which is considerably quicker but less accurate than LC-MS/MS. Preparation of the sample, extraction, hydrolysis, and determination are typically the steps in the procedure. Beloglazova et al. [57] first established an immunochemical approach for ZEA and zearalenone-4-glucoside detection in cereal samples. Hou et al. [58] developed a spherical colloidal gold-based immunochromatographic test strip that could rapidly and precisely monitor the presence of ZEA in grains with a visual detection limit of 6 ng/mL. Immunoassay is quick, high-throughput, practical, and inexpensive. However, it can only provide qualitative or (semi-)quantitative results, necessitating a follow-up confirmatory analysis [59].

Electrochemical biosensors, characterized by high sensitivity, selectivity, cost-effectiveness, and simplicity, are extensively applied for ZEA detection. In addition to direct identification, the recognition elements of electrochemical approaches frequently include antibody, aptamer, and dsDNA, with antibodies and aptamers being the most common receptors [55,60,61,62]. The majority of electrochemical ZEA biosensors are electrochemical immunosensors based on high affinity interactions between antigens and antibodies [63]. The electrochemical immunosensors were applied to multiple cereals and cereal-based samples, including maize, wheat, and others, spanning a broad range of detection from ng/mL to mg/mL. Aptamers are chosen over antibodies for ZEA detection due to their superior sensitivity and specificity, as well as their ease of synthesis, regeneration, and chemical modification [55]. Ji et al. [61] proved that an aptamer-based electrochemical biosensor has a detection range of 1 fg/mL to 100 ng/mL. Currently, there are two aptamers for ZEA determination, and interference from complex dietary matrices poses the greatest challenge for molecular diagnostic components [55]. With additional advancements in surface immobilization and aptamer-based biosensors, electrochemical biosensors are projected to become a more reliable analytical platform for ZEA and masked ZEA detection.

## 5. Fate of ZEA in Cereal-Based Food Processing

In addition to field and storage contamination, food processing plays a crucial role in ZEA transformation and content fluctuation. This section focuses primarily on the fate of ZEA throughout the processing of various typical cereal-based foods, including bread, biscuits, cake, pasta, and beer, in order to provide a thorough explanation of ZEA alterations and a reference for maintaining the safety of cereal-based foods.

### 5.1. Bread

**Figure 2:**
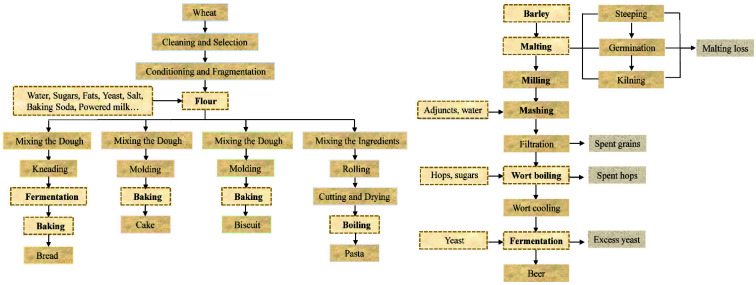
Steps of wheat and beer processing. Steps affecting zearalenone content during food processing were marked with dotted line.

After the mixed dough has been prepared, the remaining steps in bread processing include kneading, fermentation, and baking (Figure 2). Existing literature has proved that fermentation and baking are the key determinants of ZEA content changes during the bread-making process. To replicate the natural contamination of ZEA, artificially contaminated flour is commonly employed. The majority of studies confirmed that fermentation induced ZEA reduction in bread production. A meta-analysis that compiled research from 1983 to 2017 revealed that dough fermentation could reduce ZEA level by around 3% [64]. Heidari et al. [65] similarly discovered lower ZEA levels in flour following fermentation, with a higher decrease in the first proof due to the faster growth of *Saccharomyces cerevisiae* (*S. cerevisiae*). Yeasts involved in fermentation may create an acid environment that promotes the transformation and degradation of ZEA. Therefore, the type of yeast used during fermentation is crucial for ZEA reduction. Compressed yeast was considerably more effective than instant dry yeast under identical processing circumstances [65]. The higher destruction of yeast may be due to two aspects. The first is that it can produce more CO_2_, resulting in a reduction in pH, the development of various destructive substances, such as acids, alcohols, and enzymes, and a prolonged fermentation period. The second is the performance of yeast cell wall adsorption. It was proven that the adsorption capacity of yeast depends on the mycotoxin concentrations [66]. This concentration-dependent relationship was also observed in the bread-making process, where the initial ZEA concentration in the dough was proportional to the decrease efficiency of *S. cerevisiae* [67]. However, Cano-Sancho et al. [68] reported no statistical difference in ZEA levels between the dough and fermented dough at 25 °C in wheat bread production, which may be attributable to the initially low ZEA contaminated level of wheat flour (0.319 ± 0.281 μg/g).

Concerning the baking process in bread production, changes in ZEA content are controversial. Bol et al. [69] examined ZEA levels in flour and bakery products after thermal processing and pointed out that ZEA decreased by about 89% after baking at 220 °C for 35 min, indicating that heat treatment was effective for ZEA reduction. Heidari [65] declared temperature and processing time as the most important factors in declining ZEA, provided that ZEA conjugates were formed during bread production. Gilbert et al. [70] suggested that around 40% of ZEA was lost after bread baking when the dough was contaminated with a high level of ZEA. However, other researchers presented that heat treating failed to reduce ZEA concentration, and even increased it [12,71]. Cano-Sancho et al. [68] determined the constant ZEA concentration after 20 min of baking at 200 °C. Results of the maize bread baking experiment by Numanoglu et al. [72] showed inconsistent results: baking at 250 °C for 70 min generated a little increase in ZEA (3.3%) in the crumb, but thermal treatment reduced ZEA by 13% in the crust. This result was also consistent with their previous research on ZEA content fluctuations in traditional Turkish maize bread [73]. Since ZEA is somewhat heat stable, it cannot be anticipated to degrade significantly after moderate thermal processing in the absence of a reaction that would produce masked ZEA. Numerous factors, such as the initial ZEA concentration of contaminated flour, baking temperature and duration, and masked ZEA formation and degradation, are hypothesized to influence ZEA concentration during the baking process. Increased masked ZEA content in bread is possible as a result of decreased ZEA level after heat treatment. Several masked ZEAs, including β-ZOL, α-ZOL, ZEA-4-sulfate, and ZEA-4-glucoside, have been identified in bread (Table 1). Alternatively, masked ZEA in flour can break down and release free ZEA when heated. Bryla et al. [74] approved that Z14G and Z14S concentrations were reduced by 42% and 48%, respectively, during fermentation and baking of malt loaf production. However, the cooccurrence of ZEA and masked ZEA, as well as their changes during food processing, remain understudied, hence increasing food safety issues. To improve hazard control, it is vital to monitor the changes in ZEA and masked ZEA at various production steps.

### 5.2. Biscuit and Cake

The ingredients used to make biscuits and cake are almost identical to those used to make bread, but unlike bread, fermentation is not a stage that is required to make biscuits and cake (Figure 2). Therefore, research on changes in ZEA concentration during the process considers the effects of ingredients and baking.

Scudamore et al. [75] studied the impact of flour content on ZEA levels in two biscuits throughout processing. It was discovered that the ZEA content of semisweet biscuits comprising 70% flour decreased by 27.5% when fat and sugar were added, whereas the ZEA content of crackers having nearly 90% flour remained unchanged after baking [75]. Following 15 min of baking at 190 to 200 °C with 3% baking powder added to the dough, ZEA levels decreased by between 16% and 27% [76]. It was stated that the dilution effects were the likely mechanisms of ZEA reduction caused by component addition [69].

Another key factor in the production of cakes and biscuits that affects changes in ZEA level is baking. A previous study proved that baking led to 93% and 84% ZEA loss during the production of cake and biscuits, respectively, and that a higher baking temperature of 270 °C was more conducive to ZEA reduction than a lower temperature of 170 °C [69]. However, ZEA reduction cannot be achieved alone with heating treatment. In a different study conducted by Bol et al. [69], the most substantial drop of ZEA occurred in cakes with a high flour proportion of 75%, and it was found that liposoluble ZEA was probably easier to solubilize in cakes with a high oil content and low amount of water, hence promoting ZEA decomposition in baking. Furthermore, the addition of either ammonium persulfate (0.03%) or kansui (1% potassium carbonate) considerably accelerated the degradation of ZEA during heating [76].

In conclusion, these results suggested that biscuit and cake ingredients play a significant influence in ZEA level variations, and that baking led to ZEA reduction in biscuit and cake processing. It is still unclear, though, how adding substances such as oil, sugar, and baking powder reduced the ZEA concentration. The thermal treatment of bread, biscuits, and cake had comparable effects on ZEA concentrations. Although no study has reported the presence of masked ZEA in biscuit and cake, it is probable that during the baking process, ZEA reacts with other components to generate masked ZEA in dough. To further reduce masked ZEA contamination, understanding of masked ZEA occurrence in biscuit and cake processing is necessary.

### 5.3. Pasta

The preparatory stages for producing pasta resemble those for preparing bread, cake, and biscuits (Figure 2). Before being brought to market, the mixed dough is rolled, cut, and dried. The operations of rolling and cutting influence the shape of pasta, while drying reduces its moisture content. While boiling was discovered to promote ZEA reduction in pasta, no studies have shown that rolling or drying during the pasta-making process influences the level of ZEA. Therefore, this section focuses mostly on the impact of boiling on ZEA alterations of pasta.

Bol et al. [69] found that ZEA significantly decreased by 75% after 15 min of cooking, with around 10% leaching into the cooking water. Adding 1% potassium carbonate to instant noodles spiked with 1 and 20 μg/kg ZEA and heating at 100 °C for 3 min, followed by 140–150 °C for 2 min, degraded ZEA by approximately 48% and 62%, respectively [76]. Boiling is effective for ZEA reduction, and the decomposition efficiency appears to be dependent on the initial ZEA level and temperature. Similar to the bread-making process, pasta with a higher initial ZEA contamination concentration may see more toxin reduction after boiling. However, compared to bread, biscuit, and cake, pasta requires a different heating mode and temperature. Cooking pasta in boiling water required a temperature between 85 and 98 °C, whereas baking bread in an oven required a much higher temperature, such as 220 °C. Considering the thermal stability of ZEA, it is possible that the mycotoxin is more stable in boiling than in baking. To maintain the safety of pasta, it is important to ensure the quality of the ingredients, which is the primary source of ZEA contamination. In addition, more research is required to better understand the mechanisms underlying ZEA level variations and masked ZEA incidence during the drying and boiling processes.

### 5.4. Beer

The primary ingredient in beer is barley, along with water, malt, hops, and various additives such as maize, oats, and sorghum. ZEA can easily contaminate the raw materials of beer, hence increasing the likelihood of beer contamination. Recent studies have shown that some brewing processes affect ZEA and masked ZEA levels. Figure 2 illustrates the various steps involved in brewing beer.

The malting process includes germination and kilning. To encourage germination, barley is steeped for 36–52 h at 12–20 °C, immersed in aerated water, and exposed to humified air to increase the moisture content to approximately 45%. A further kilning is intended to reduce the moisture content of germinating barley to roughly 4~5%. Plentiful chemical reactions occur during the kilning step, which has a significant impact on the color, odor, flavor, and texture of the beer. Piacentini et al. [77] found that ZEA concentrations on the third day of germination were greater than those on the first day of steeping, indicating that ZEA production was greater during germination. Due to ZEA conversion to α-zearalenol, β-zearalenol, and other masked toxins, the level of ZEA in the malting process was reduced by around 79% [77]. Pascari et al. [78] discovered that following a 40% loss of ZEA in the first steeping water, the ZEA level in barley increased during the kilning process, leading to a constant ZEA concentration in the malting stages. Similarly, another study demonstrated the same variations in ZEA levels during the steeping process, but the ZEA content rose during germination and kilning [79]. El-Banna [80] found that malting might severely degrade ZEA, although Wall-Martínez et al. [81] asserted that malting had no effect on ZEA. Since barley is vulnerable to scab and is easily contaminated with ZEA, the initial ZEA concentration controls the volatility of ZEA level throughout malting. Tabuc et al. [82] investigated ZEA concentration in 21 malting barleys in the southeastern part of Romania and found that ZEA contaminated nearly 71.4% of barleys, with an average concentration of 132.7 μg/kg. ZEA in contaminated barley would likely be released during the malting process, resulting in an increase in ZEA concentration. Alternatively, ZEA may change into α-zearalenol, β-zearalenol, and other masked toxins during malting, resulting in a decrease in ZEA.

The malted barley was then milled and mashed, and some additive adjuncts were added (Figure 2). After filtration, the wort was boiled at a high temperature, and most ZEA decreased during the process. More than 89% of ZEA was shown to be removed by mashing and boiling [81]. According to a study by Pascari et al. [83], 30 min of boiling resulted in a 100% reduction in ZEA. Longer boiling time promoted ZEA degradation but may diminish nutritional quality [84]. High heat tolerance of ZEA and the inclusion of adjuncts such hops, corn, wheat, and sorghum might result in stable or increased ZEA content after boiling [77,85]. Changes in the ZEA concentration during fermentation are likewise contentious. GILBER [70] proposed that ZEA remained constant during fermentation, whereas Wall-Martínez et al. [86] found that 30~70% of ZEA was degraded following fermentation in commercial beer due to yeast cell adsorption. A study revealed that ZEA in wort varied from 26 to 285 μg/L and from 20 to 201 μg/L in beer, with a carry-over ranging from 23 to 403% [87]. The high carry-over could be ascribed to the addition of unfermented wort after fermentation, leading to an increased ZEA level. In addition, the transformation of ZEA to masked ZEA during fermentation may contribute to the decrease in ZEA concentration. Scott et al. [88] confirmed that in the first 1–2 days of fermentation, *Saccharomyces cerevisiae* metabolized 69% of ZEA to β-zearalenol and 8.1% of ZEA to α-zearalenol. It was observed that 85.9% of ZEA was converted to β-zearalenol in artificially ZEA-contaminated fermented wort [89]. Chilaka et al. [90] found that α-zearalenol and β-zearalenol were present in beer at concentrations of 22 ± 18 and 31 ± 16 μg/kg, respectively, although masked ZEA did not contaminate raw sorghum.

In conclusion, these results suggested that four steps (adding raw material and adjuncts, malting, boiling and fermentation) in the beer-making process significantly affect the occurrence of ZEA and masked ZEA in beer. The most important factor is the level of ZEA contamination in the raw materials and adjuncts, which determines the presence of ZEA and masked ZEA in the final product. Malting and fermentation promoted ZEA transformation to masked forms. However, during these processes, complex biochemical reactions occur, resulting in the controversial statement regarding ZEA content fluctuations. Due to the thermal stability of ZEA and the addition of adjuncts, variations in ZEA concentration during boiling are unexpected. To minimize masked ZEA development as well as nutritional and flavor loss, it is necessary to conduct additional research on the fate of ZEA in the beer-making process.

## 6. Allowable Limit of ZEA in Cereal and Cereal-Based Food

The maximum limit of ZEA in cereal and cereal-based food has been established in various countries (Table 2). The allowable residue levels of ZEA vary greatly from country to country. The European Union set the maximum residue levels for wheat at 100 µg/kg, however, in Armenia, Colombia, Russia, and other countries, the maximum residue levels reached 1000 µg/kg. The difference may be attributed to the availability of toxicological data, information on dietary exposure, the distribution of mycotoxins across products, the regulations of other nations with which there are trade contacts, and the availability of analytical methods [91]. However, several nations have neither specified maximum levels nor guidance limits for ZEA in cereals and cereal-based food. In addition, the presence of masked ZEA was excluded from the regulation. Considering the incidence of both ZEA and masked ZEA, it is suggested that future legislation incorporate masked ZEA.

## 7. Conclusions

In conclusion, ZEA is a prevalent mycotoxin in cereals, but little data have reported the presence of masked ZEA (particularly phase II metabolites) to date. The co-occurrence of ZEA and masked ZEA in cereals has received little attention. During the processing of cereal-based foods, certain operations resulted in ZEA level variations and masked ZEA formation. Co-contamination of ZEA and masked ZEA is of particular concern due to potential additive or synergistic health effects. Regarding this topic, the primary future challenge includes: (1) increasing the number of analyzed masked ZEA in cereals; (2) improving knowledge on the effects of cereal-based food processing on the incidence of ZEA and masked ZEA; (3) developing rapid and precise methods for simultaneous detection of ZEA and masked ZEA; (4) risk assessments for the ZEA and masked ZEA that are ordinarily regarded as negligible; (5) revising the legislative limits of masked ZEA to ensure the greatest protection of human health.

## Figures and Tables

**Table 1:** Occurrence of masked ZEA in cereals and cereal-based food.

Masked ZEA	Cereal and Cereal Based Food	Incidence (%)	Mean (µg/kg)	Ref.
β-ZOL	Wheat	10.0	3.5	[48]
	Wheat	16.7	49	[31]
	Wheat	44.4	12	[49]
	Wheat	100	7	[49]
	Wheat	44.4	6	[49]
	Maize	100	11	[14]
	Maize	66.7	42.5	[31]
	Oats	32.3	3.0	[48]
	Oats	16.7	46	[31]
	Millet	100	16	[14]
	Sorghum	95	27	[14]
	Barley	2.9	2.0	[48]
	Bread	50	79	[31]
	Cornflakes	66.7	53.3	[31]
α-ZOL	Maize	100	27	[14]
	Maize	100	96.8	[31]
	Wheat	13.3	0.6	[31]
	Wheat	11.1	8	[49]
	Oats	9.7	1.9	[48]
	Oats	33.3	59.5	[31]
	Sorghum	100	8	[14]
	Millet	100	18	[14]
	Barley	2.9	0.6	[48]
	Bread	33.3	64	[31]
	Cornflakes	33.3	30	[31]
ZEA-4-sulfate	Maize	16.7	51	[31]
	Wheat	33.3	11	[31]
	Oats	16.7	12	[31]
	Bread	16.7	24	[31]
ZEA-14-sulfate	Barley	8.8	10.6	[48]
	Oats	29.0	31.6	[48]
	Wheat	40.0	4.9	[48]
ZEA-16-glucoside	Barley	23.5	<LOQ	[48]
	Oats	58.1	4.2	[48]
	Wheat	6.7	2.1	[48]
ZEA-14-glucoside	Barley	17.6	2.7	[48]
	Oats	3.2	<LOQ	[48]
	Wheat	6.7	0.6	[48]
ZEA-4-glucoside	Maize	16.7	274	[31]
	Bread	33.3	20	[31]
β-ZOL-4-glucoside	Maize	50	152	[31]
	Oats	16.7	20	[31]
α-ZOL-14-glucoside	Barley	23.5	2.9	[48]
	Wheat	16.7	3.1	[48]
α-ZOL-4-glucoside	Maize	16.7	283	[31]
β-ZOL-14-glucoside	Barley	2.9	0.7	[48]

**Table 2:** Maximum limits for zearalenone in cereals and cereal products in various countries.

Regulatory Bodies	Cereal and Cereal-Based Food	Maximum Levels (µg/kg)	Ref.
European Union	Wheat	100	[92]
	Corn	350	
	Cereal, cereal flour, and bran for direct consumption	75	
	Corn flour	200	
	Bread and other bakeries	50	
	Cereal snacks	50	
	Corn-based snacks	100	
Brazil	Wheat flour, pasta, crackers and bakery products, cereals and cereal products (except wheat), and malted barley	100	[93]
	Processed rice and derivates	100	
	Brown rice	400	
	Rice bran	600	
	Corn-based products	150	
	Whole wheat, whole wheat flour, wheat bran	200	
	Corn and wheat in grains	400	
China	Wheat, wheat flour	60	[94]
	Corn, corn flour	60	
Australia	Cereals	50	[95]
Chile	All foods	200	[95]
Armenia	All foods	1000	
Belarus	Barley, wheat, maize	1000	
Colombia	Sorghum	1000	
Indonesia	Maize	Not detectable	
Iran	Barley	400	
	Maize, wheat, rice	200	
Moldova	Wheat and wheat flour, barley and barley flour, maize and maize flour	1000	
Morocco	Cereals	200	
Russia	Wheat, barley, maize, corn	1000	
Serbia and Montenegro	Corn	1000	
Ukraine	Grains, beans, sunflower press, flour, bread, all nuts, all seeds to be used for immediate human consumption and for processing into the products for human consumption, wheat middlings	1000	
Uruguay	Corn, barley	200	

## Data Availability

Not applicable.
